# Identification and Clinical Correlation of Circulating MAIT, γδ T, ILC3, and Pre-Inflammatory Mesenchymal Cells in Patients with Rheumatoid Arthritis and Spondyloarthritis

**DOI:** 10.31138/mjr.251022.iac

**Published:** 2023-09-25

**Authors:** Maria Kyriakidi, Eleni-Kyriaki Vetsika, Georgios E. Fragoulis, Maria Tektonidou, Petros P. Sfikakis

**Affiliations:** 1 Centre of New Biotechnologies and Precision Medicine (CNBPM) School of Medicine, National and Kapodistrian University of Athens, Athens,; 2 First Department of Propaedeutic Internal Medicine, National and Kapodistrian University of Athens, “Laiko” General Hospital, Athens

**Keywords:** rheumatoid arthritis, psoriatic arthritis, axial spondyloarthritis, immuno-profiling, circulating mesenchymal cells, biomarkers, mass cytometry

## Abstract

Inflammatory rheumatic diseases (IRDs), such as rheumatoid arthritis (RA) and spondyloarthropathy (SpA), comprise a heterogeneous group of immune-mediated disorders, characterised by the presence of localised and/or systemic inflammation. The limited knowledge of the pathogenesis and the complex mechanisms involved in the induction and maintenance of inflammation in IRDs have impeded the development of reliable biomarkers and the discovery of new therapeutic targets. Although the involvement of heterogeneous cell populations in the pathogenesis of IRDs has been recognised, the characterisation of these cellular subsets in the peripheral blood of patients has not been studied yet. Mass cytometry, allowing the simultaneous detection of more than 120 different parameters in single-cell resolution, will enable the identification of circulating cell subpopulations that might play a pivotal role in IRDs pathophysiology and their potential use as therapeutic targets.

## INTRODUCTION

Rheumatoid arthritis (RA) is a chronic, inflammatory autoimmune disease with a prevalence rate ranging from 0,5 to 1% of the general population, differing between ethnicities, with women being two to three times more likely to develop RA compared to men.^1^ RA predominantly affects the joints and is associated with autoantibodies against immunoglobulin G and citrullinated proteins. Based on the presence or absence of these autoantibodies, RA patients can be subdivided into seropositive and seronegative, respectively.^2^ The clinical manifestations of RA include typically symmetrical joint swelling, redness, and arthralgia, with morning stiffness and severe motion impairment in the involved joints.^3^ Several risk factors are involved in the development of RA, with genetics playing a pivotal role and environmental factors, including smoking, triggering the disease in the case of genetically susceptible individuals.^1^

Spondyloarthritis (SpA) comprises a group of chronic inflammatory rheumatic diseases characterised by inflammation of sacroiliac joints, peripheral joints, and the spine, with various clinical manifestations.^4^ This group includes ankylosing spondylitis (AS), reactive arthritis, psoriatic arthritis (PsA), arthritis associated with inflammatory bowel disease, and undifferentiated SpA.^5^ Axial spondyloarthritis (AxSpA), including AS, is a condition causing inflammation in the spine and/or the sacroiliac joints and can be subdivided into radiographic and non-radiographic SpA, based on the ability to detect joint damage with X-rays.^6^ The most common clinical manifestations are chronic back pain and spinal stiffness, however peripheral and extra-musculoskeletal symptoms can occur too.^6^ Regarding peripheral spondyloarthritis (pSpA), the most common subset is PsA, a disease that affects approximately 30% of patients with skin psoriasis, with an equal prevalence between men and women. PsA involves diverse clinical manifestations, including peripheral and axial arthritis, enthesitis, dactylitis, skin and nail damage, as well as extra-musculoskeletal symptoms, such as intestinal inflammation and anterior uveitis.^7,8^ Among the risk factors that have been suggested to be implicated in the development of PsA are genetics, immunological and environmental factors, including physical trauma, obesity, and smoking.^7^

The pathophysiology of inflammatory rheumatic diseases (IRDs) involves a complex interplay between multiple cell types, including leukocyte populations, synovial fibroblasts, chondrocytes, osteoclasts, and others.^3,5–7^ In patients with RA, researchers discovered infiltrates of cells of both adaptive and innate immunity, such as T cells, B cells, macrophages, monocytes, neutrophils, and plasma cells.^3,9,10^ Regarding SpA, the role of the IL-23/-17 axis has been recognised over the last few years.^11^ Although IL-17 was initially thought to be produced solely from Th17 cells (triggered by IL-23), recent studies have highlighted the important role of rare cell subpopulations like MAIT, ILC3, and γδ T cells. These cells seem to produce IL-17 irrespective of IL-23 and their frequency and characteristics seem to differ between the different clinical expressions of SpA.^12,13^ However, none of these studies have investigated the presence of the subpopulations of these cells in the periphery.

In addition to leukocytes, other cell types appear to play an active role in the pathogenesis of RA, such as pre-inflammatory mesenchymal (PRIME) and fibroblasts. Our group proposed an essential role of cadherin-11 in joint inflammation and fibrosis. Cadherin-11 mRNA transcripts have been identified in the peripheral blood, reflecting viable cells in patients with RA and systemic sclerosis (SSc). Cadherin-11 transcripts found in circulation are associated with established disease and the presence of polyarthritis in RA, as well as with diffuse skin involvement in SSc.^13,14^ Moreover, preliminary data from our group showed that rare circulating mesenchymal cells can be identified in the peripheral blood of RA and PsA patients using mass cytometry and might be of pathogenetic importance.^15^ Recently, Orange et al. have identified in the circulation the PRIME cells which are the precursors to inflammatory sublining fibroblasts.^16,17^

Researchers have found that these cells express classical mesenchymal and synovial fibroblast surface markers and their presence is associated with the onset of disease outbreaks.^17^ Both studies proposed that these cells traffic in the peripheral blood and subsequently migrate to the synovium, contributing to the inflammatory process. Synovial fibroblasts also play a crucial role in the pathogenesis of SpA, especially of PsA, however, the presence of the PRIME cells in the periphery of these patients has not yet been examined. Therefore, the identification of circulating cell populations and their correlation with clinical data can help us discover cell patterns of cell types that might be used as indicators of activity and predictors of exacerbations of RA and SpA, but also as indicators of response to treatment.

Mass cytometry (Cytometry by Time-Of-Flight, CyTOF), is an advanced technology that allows the simultaneous detection of more than 120 different parameters in single-cell resolution, thus being a valuable weapon in the quiver of clinical doctors and researchers to discover new cells and biomarkers, valuable for diagnosis and response to treatment of various diseases.^18^ Leite Pereira et al., using CyTOF technology, characterised the immunological profile of patients with RA. They identified two potential new blood subpopulations of neutrophils (CD11b^low^CD16^high^) and T-cells (CD11a^high^ Granzyme Bhigh), which could be involved in RA pathology.^19^ However, the exact role of these subpopulations has not been studied yet. Moreover, Zhang et al., using synovial tissue from patients with RA or osteoarthritis (OA), analysed CD45-Podoplanin^+^ cells with mass cytometry and found eight putative cell clusters with differential protein levels of CD90, CD34, and cadherin-11.^20^ However, this finding has not been explored in the peripheral blood. Furthermore, Koppejan et al., analysing peripheral blood mononuclear cells (PBMCs) from early untreated RA patients using mass cytometry, tried to identify differences in immune cell subsets between ACPA-positive and ACPA-negative RA patients. Despite finding no differences in major immune lineages, they identified a reduced population of innate cells with an activated basophil-like phenotype in ACPA-negative patients, with the possible role of these cells in the immune response associated with RA remaining unclear.^21^ Understanding the phenotype and mechanisms of each cell type in IRDs permits a deeper insight into these complex syndromes. Therefore, the use of mass cytometry (high-dimensional single-cell profiling) can help us create cell signatures in the periphery concerning both RA and SpA and reveal new cell subsets specific to these diseases and ideal for therapeutic targeting.

## AIMS OF THE STUDY

This investigation aims to define the heterogeneity of circulating cells in patients’ samples with IRDs, including RA and SpA, and to correlate this information with the disease state and activity.

For this purpose, this research will use peripheral blood in order:

1. To characterise the heterogeneity of circulating immune cells by performing deep immune cell profiling;
2. To investigate the presence and levels of circulating rare cells-expressing surface markers of synovial fibroblasts and mesenchymal markers such as cadherin-11, podoplanin, CD90, and Notch3;
3. To create signatures of heterogeneity between disease diagnoses and phenotypes;
4. To define whether a particular pattern of cell types can be used as a predictive biomarker for disease progression and response to treatment.

## RESEARCH METHODOLOGY

### Study design and inclusion criteria

The study will be conducted in a cohort of 50 patients with active RA, PsA, or axSpA and 13 healthy donors. Patients will be enrolled from the Rheumatology Unit, First Department of Propaedeutic and Internal Medicine, Athens University Medical School, Greece. The study complies with the Ethical Principles for Medical Research Involving Human Subjects according to the World Medical Association Declaration of Helsinki and the Oviedo Convention and has been approved by the local Ethics and Scientific Committees of the University Hospitals of National and Kapodistrian University of Athens. All patients will be treated in the context of standard clinical practice and according to national and international guidelines. The main inclusion criteria will be:

1. Patients (consecutive) with active: 1. RA (RA 2010 criteria),
2. PsA (CASPAR criteria),
3. axSpA (ASAS criteria), regardless of treatment;
4. Signed informed consent form.

The main exclusion criteria will be:

1. Patients with coagulation disorders or treated with anticoagulants;
2. Patients who have received rituximab (anti-CD20 antibody) in the last 6 months;
3. Patients with active neoplasm (solid or haematological) or who have received chemotherapy in the last 6 months.

### Blood Samples

10 ml of peripheral blood from patients and healthy donors will be obtained and stored in ethylenediaminetetraacetic acid (EDTA) tubes for the evaluation of different subsets of circulating cells by high-dimensional mass cytometry. No other intervention will be performed, and clinical data will be collected from the patient’s file, as recorded during their regular visit to the Rheumatology Unit. Sample collection for each patient will be performed at baseline and three months after treatment.

### Deep peripheral blood circulating cells profiling

The presence and the levels (percentages and absolute numbers) of the different cell subtypes will be assessed using a panel of 36 monoclonal antibodies and will be analysed by mass cytometry. Whole blood will be stained using the Fluidigm Maxpar Direct Immune Profiling Assay (201334) panel, which contains 30 anti-human heavy metal isotope-conjugated monoclonal antibodies, and 6 additional anti-human monoclonal antibodies: anti-Notch3 (MHN3-21) ^165^Ho (3165006B), anti-CD279/PD-1 (EH12.2H7) ^175^Lu (3175008B), anti-CD90 (5E10) ^159^Tb (3159007B), anti-CD34 (581) ^110^Cd (custom), anti-cadherin-11 (16G5) ^169^Tm (custom) and anti-podoplanin (NC-08) ^142^Nd (custom). All antibodies are purchased from Fluidigm, Standard BioTools Inc., San Francisco, CA, USA. Whole blood will be stained with the antibodies, and, after red blood cell lysis, samples will be fixed and then stained with Cell-ID Intercalator-Ir (201192A) according to Fluidigm’s recommended protocols. After being prepared for acquisition, samples will be acquired using a 3^rd^ generation Helios mass cytometer. The generated FCS files will be normalised and uploaded to Cytobank (Beckman Coulter Life Sciences, Indianapolis, IN, USA) and Maxpar Pathsetter 3.0 (Fluidigm) for analysis. All the experiments will be performed in the Centre of New Biotechnologies and Precision Medicine (CNBPM).

### Statistical Analysis

Because of the observational nature of the study, it is not possible to propose a statistical hypothesis for the estimation of the appropriate sample size of the study. Statistical analysis will be performed using GraphPad Prism version 9.0 (GraphPad Software, San Diego, CA, USA). Comparisons of the percentages of the different cell populations between the different groups and subgroups at baseline will be performed using an unpaired t-test or Mann-Whitney U test. Comparisons of the percentages of the different cell populations between the two time points will be performed using paired t-test or Wilcoxon test. Qualitative factors will be compared by Pearson’s chi-squared test or Fisher’s exact test. Statistical significance will be considered at p-value < 0.05 and for two-sided tests. Correlation coefficients between the variables will be calculated according to Spearman’s rank correlation coefficient (rho).

## STUDY APPROVAL

The study has been approved by the local Ethics and Scientific Committees of the University Hospitals of National and Kapodistrian University of Athens (Protocol Number: 314/2021).
